# Do Individual Differences in Cognition and Personality Predict Retrieval Practice Activities on MOOCs?

**DOI:** 10.3389/fpsyg.2020.02076

**Published:** 2020-08-18

**Authors:** Daniel Fellman, Alisa Lincke, Bert Jonsson

**Affiliations:** ^1^Department of Applied Educational Science, Umeå University, Umeå, Sweden; ^2^Department of Computer Science and Media Technology, Linnaeus University, Växjö, Sweden

**Keywords:** retrieval practice, test-enhanced learning, e-learning, MOOC, personality, cognition

## Abstract

Online quizzes building upon the principles of retrieval practice can have beneficial effects on learning, especially long-term retention. However, it is unexplored how interindividual differences in relevant background characteristics relate to retrieval practice activities in e-learning. Thus, this study sought to probe for this research question on a massive open online course (MOOC) platform where students have the optional possibility to quiz themselves on the to-be-learned materials. Altogether 105 students were assessed with a cognitive task tapping on reasoning, and two self-assessed personality measures capturing need for cognition (NFC), and grittiness (GRIT-S). Between-group analyses revealed that cognitively high performing individuals were more likely to use the optional quizzes on the platform. Moreover, within-group analyses (*n* = 56) including those students using the optional quizzes on the platform showed that reasoning significantly predicted quiz performance, and quiz processing speed. NFC and GRIT-S were unrelated to each of the aforementioned retrieval practice activities.

## Introduction

Learning via Internet has increased its popularity during the past decades due to its advantages it offers with respect to flexibility of time (i.e., studying can be carried out at any time) and space (i.e., studying can be carried out anywhere). In this vein, a new concept, denoted as e-learning has arisen, which is an umbrella term that covers all aspects related to individualized instructions distributed over public or private computer networks intended to promote learning (Manochehr, 2006; Clark and Mayer, 2016). One particularly fast-growing learning format pertains to massive open online courses (MOOCs). MOOC refers to learning platforms to which an unlimited amount of students can enrol (either paid or unpaid), and access a wide range of courses materials, including additional learning resources such as interactive courses, problems sets (e.g., quizzing), and filmed lectures (Kaplan and Haenlein, 2016). The advantages with MOOCs lies in its flexibility, allowing students to take courses independently at their own pace, without being bound by time and place.

### Retrieval Practice

Along with the increased popularity in e-learning, several MOOC platforms have also started to apply features on their platforms with the purpose to boost learning outcomes. One such feature pertains to the opportunity to quiz oneself on the learned materials (van der Zee et al., 2018). A large body of evidence in experimental settings shows that self-testing of the to-be-learned material, typically denoted as *retrieval practice*, increase students’ long-term retention and transfer of knowledge to new situations (Roediger and Karpicke, 2006; Butler, 2010; Weinstein et al., 2010; Agarwal et al., 2012). Moreover, the benefit of retrieval practice over other study strategies (e.g., summaries, note-taking), often referred to *testing effects*, are typically not visible when knowledge is tested immediately after learning (e.g., Roediger and Karpicke, 2006). Rather, the effects are prominent over lengthier retention intervals, for instance, when students are tested a week after the learning phase (Karpicke and Roediger, 2007). Although the effects of retrieval practice on memory retention occur independently on feedback (Roediger and Butler, 2011), the inclusion of feedback strengthens learning and provide a formative component through which students can monitor their accuracy and thus prevent that erroneous learning (Roediger and Marsh, 2005). The mechanisms underlying retrieval practice remains unclear (see Rowland, 2014 for an overview), but the effectiveness has been studied and confirmed in different experimental settings, educational contexts, across a range of materials and by brain imaging studies (Dunlosky et al., 2013; van den Broek et al., 2016; Adesope et al., 2017). Thus, retrieval practice is relatively well-established and that the act of retrieving memory from information seems to strengthen memory and to reduce forgetting (Kornell et al., 2011; Rowland, 2014). The features of self-testing with feedback appears to be a very promising study technique, especially for MOOCs, as the content, typically is directed to lifelong learning (van der Zee et al., 2018). In this vein, the present study set out to test how individual differences in cognition and personality is associated with retrieval practice activities on a MOOC platform targeted for university students, providing new insights to the body of research within teaching and learning across different environments in the digital age (explicit link to the special issue: https://www.frontiersin.org/research-topics/12111/assessing-information-processing-and-online-reasoning-as-a-prerequisite-for-learning-in-higher-educ).

Retrieval practice is typically implemented in various formats on MOOCs using quizzing with and without the support of images and video clips, utilizing different response formats such as multiple-choice and short and open-answer responses for boosting learning outcomes (van der Zee et al., 2018). Such quizzing features are often optionally implemented, that is, students can complete quizzes as an additional support for their learning. However, several studies indicate that quizzes remain highly unutilized when they are optional in online course (Olson and McDonald, 2004; Kibble, 2007; Carpenter et al., 2017; Corral et al., 2020). Corral et al. (2020), for instance, showed that nearly 45% of students did not complete a single quiz during an online course covering introductory psychology and that 88% of these quizzes were not completed. Furthermore, Carpenter et al. (2017) examined the quizzing frequency among the students that took an online biology course, showing that about 50% of the students completed the practice quizzes that were made available. However, the findings reported above have been observed at the group level, and it is still unclear whether individuals with certain traits are more likely to engage in quizzing than others are, thus prompting further research.

Besides that quizzing remains largely unexploited by students, it is also unclear in what way, and in what volume the quizzes are used by those individuals that actually engage in retrieval practice on MOOC platforms. As previously mentioned, the majority of previous retrieval practice studies on this topic are experimental, typically applied in the context of laboratory or classroom settings (for a meta-analysis, see Adesope et al., 2017), whereas only a few ones have focused on examining how retrieval practice on MOOC platforms are related to learning outcomes (Davis et al., 2016, 2018). Davis et al. (2016) examined whether students’ learning outcomes on a MOOC functional programming course would be altered if the participants (*n* = 2166) were prompted with retrieval practice cues following each lecture. Compared to a control group receiving no quizzes, the results showed no beneficial effects of retrieval practice neither in test performance or actual course grades. In another study, Davis et al. (2018) prompted participants to write summaries of the content following each video clip on a MOOC course on Coursera. The results showed that the amount of written summaries were associated with a better performance in the weekly quiz assessments, but not in a better performance in the final course exam.

Albeit retrieval practice has been extensively examined, few studies have focused on for which individuals this learning technique is beneficial. Indeed, there are large inter-individual differences in most of human-related behavior, but one background factor that consistently has shown to influence learning outcomes is cognitive ability. Study results show that fluid intelligence (i.e., the ability to solve problems in novel situations) and working memory (i.e., the ability to maintain and manipulate information over a short period before it decays) are both reliable predictors of academic attainment (Turner and Engle, 1989; Cowan et al., 2005; Krumm et al., 2008; Furnham and Monsen, 2009; Ren et al., 2015). The few studies examining cognition in relation to retrieval practice have been somewhat mixed, with some studies showing that cognitively strong individuals show greater testing effects (Tse and Pu, 2012; Agarwal et al., 2017), especially when prompted with more difficult items (Minear et al., 2018), or results that support effects in neither direction (Brewer and Unsworth, 2012; Wiklund-Hörnqvist et al., 2014; Bertilsson et al., 2017). With respect to cognitive ability and retrieval practice on MOOCs, the evidence is scarce, albeit one study investigating this relationship shows that better cognitive ability is associated with higher accuracies in quizzes, and tend to spend more time on quizzing themselves (Fellman et al., 2020).

Besides cognitive abilities, personality characteristics are important for learning outcomes as well. Especially on MOOCs where quizzing is optional, it is plausible to assume that individual characteristics tapping on motivation, openness and curiosity for learning new things are important traits for maximizing the utility of the platform. One personality trait shown to be important for learning is “the tendency to engage in and enjoy thinking”. This ability is typically referred to as *need for cognition* (NFC; Cacioppo and Petty, 1982). Individuals with high NFC typically analyze and seeks to understand information and events in their surroundings, whereas low NFC individuals are more likely to rely on experts or cognitive heuristics. Hence, high NFC individuals typically approach problem-solving tasks more positively than those with low NFC (Cacioppo et al., 1996). In traditional classroom settings, high NFC has been found to result in better performance when solving math problems (Dornic et al., 1991), and to predict academic performance (Sadowski and Gülgös, 1996). With respect to retrieval practice in experimental settings, NFC appears to be weakly related to recall performance in quizzes (Bertilsson et al., 2017; Stenlund et al., 2017), but to our knowledge, no previous study has specifically examined the relationship between NFC and retrieval practice activities on MOOCs.

Another personality trait that has been shown to be critical for learning outcomes is the perseverance and passion for long-term goals. This ability, denoted as *GRIT* (Duckworth et al., 2007), has shown to be a reliable predictor for several important outcomes such as academic achievement and life success (Duckworth et al., 2007; Eskreis-Winkler et al., 2014). It has been suggested that GRIT contribute uniquely to learning outcomes as it works independently of intelligence, and that both talent and GRIT is necessary to become highly competent in a specific skill (Duckworth et al., 2007). To our knowledge, only one study has examined the relationship between GRIT and retrieval practice in educational classroom settings (Bertilsson et al., 2017). In that study, the authors conducted two between-subjects design experiments where Swedish participants were to learn novel Swahili words either in a re-study condition or in a retrieval practice condition. While both experiments showed that those receiving retrieval practice outperformed those receiving re-study in recall following 4 weeks, the results showed no evidence that NFC would have any moderating role in these gains. However, to our knowledge, no study has investigated GRIT in relation to retrieval practice activities on MOOC platforms, deserving further scrutiny.

Lastly, it is worth pointing out that both NFC and GRIT are personality characteristics suggested to be stable over time and thus influence learning (Duckworth et al., 2007; Stenlund and Jonsson, 2017). Within the context of students using MOOC’s platforms where the student has a greater autonomous responsibility for his/her studies, personality factors are potentially even more critical. Hence, the use of these platforms are often (as in the present study) not mandatory for the students, and as shown by Corral et al. (2020), the majority of students do not complete their online quizzing. Potentially, the likelihood of using retrieval practice in MOOC’s platforms is associated with personality characteristics.

## Materials and Methods

### Study Design

The research question probed for in the present study was: Are individual differences in cognitive ability, and personality characteristics are related to retrieval practice activities on a MOOC platform? The data for this study stems from an interactive MOOC platform in Sweden titled Hippocampus (see https://www.hypocampus.se). Approximately 15 000 students use Hippocampus as a fee-based platform (99 Swedish SEK/month; approximately $10.49/month) for carrying out university courses, with most of the users consisting of medical students. The MOOC platform provides the students with compressed course materials that are highly relevant for the to-be-completed courses at their universities. Specifically, instead of completing the course by reading from the course books, the content of the course is transferred to the interactive MOOC platform. Hippocampus also provides a high degree of learner control, offering more than 50 interactive courses covering different topics in medicine that students can complete non-linearly at their own pace (i.e., they can choose to jump back and forth from a course to another). With most relevance for the present study, the students also have the optional possibility to quiz themselves on the materials they just read, building upon the principles of *retrieval practice* (Dunlosky et al., 2013). These optional quizzes are implemented at the end of each learning section. Altogether 105 university-dwelling participants that were carrying out studies on the MOOC took part in this study. Cognitive ability among the participants was measured with the Raven’s Advanced Progressive Matrices (RAPM; Raven et al., 1991). For measuring the tendency to engage in and enjoy thinking, and the perseverance and passion for long-term goals, participants were assessed with the questionnaires Need For Cognition (NFC; Dornic et al., 1991), and the Short Grit Scale (GRIT-S; Duckworth and Quinn, 2009), respectively.

The relationship between individual characteristics (i.e., cognitive ability, personality) and retrieval practice activities on the MOOC was examined in a two-fold way. First, using between-group analyses, we examined whether individuals with high usage of the optional quizzes (henceforth *high retrieval practice*; high-RP) differed from the individuals with low usage of the optional quizzes (henceforth *low retrieval practice*; low-RP) with respect to our three predictors RAPM, GRIT-S, and NFC. Second, using within-group analyses including only the high-RP group, we extracted three measures of relevant retrieval practice activities, which we presupposed that could be related to cognitive- and personality measures, and those variables were regressed on our three predictors. The three target outcomes of retrieval practice were: (1) number of quizzes taken per study session, (2) accuracy in taken quizzes and (3) quiz processing speed per study session. Note that the reason for excluding the low-RP group in the within-group analyses were justified, as this group had barely engaged in retrieval practice activities on the MOOC platform (see “Between-Group Analyses” in the Results section for more details). For the between-group analyses, we hypothesized that higher cognitive ability, as well as higher grittiness and need for cognition, would increase the likelihood for belonging to the high-RP group. For the within-group analyses, we surmised that the cognitively high-performing individuals, individuals with high GRIT-S, and individuals with high NFC would use the optional quizzes more persistently, show higher quiz accuracies, and exhibit faster reaction times in the quizzes. Our attempt to unravel individual characteristics that bear importance for retrieval practice activities on MOOCs will hopefully yield new insights to the body of research within teaching and learning across different environments in the digital age (explicit link to the special issue: https://www.frontiersin.org/research-topics/12111/assessing-information-processing-and-online-reasoning-as-a-prerequisite-for-learning-in-higher-educ).

### Data Description, Participants and Methods

Regarding the technical aspects, each day that a student login and use the MOOC (i.e., Hippocampus), a large amount of interactional data is generated. The data is collected using JavaScript methods available in the user’s browser and stored in the backend system in a database. The log-files retrieved from the database are organized into two tables: *reading_material* and *quiz_material.* The *reading_material* table contains data related to student interaction with learning materials in a course and can be used to identify reading time information (e.g., the amount of time the student was active on a particular page). The *quiz_material* table contains information regarding quiz activity such as the number of quizzes taken, and total time spent on quizzes. As this study focus solely on retrieval practice activities, only data stemming from the *quiz_material* table was analyzed. All available data from the quiz_matieral table within the date range 01.01.2019 – 02.02.2020 was extracted. Feature extraction was computed by aggregating scores as a function of a particular student (labeled as ‘user Id’ in the dataset).

The participants in the present study consisted of medical students who were studying at Hippocampus platform to prepare themselves for the actual exam at their university. The study was approved by the Regional Vetting Committee (2017/517-31), Sweden, and informed consent was obtained from all participants. All students on Hippocampus were invited to complete the test session consisting of a background questionnaire, personality questionnaires, and a reasoning task capturing cognitive ability^1^. The test session was administered online using an in-house developed web-based test platform by sending a link to the students via email (i.e., the participants could complete the experiment on a computer of their choosing) (Röhlcke et al., 2018; Fellman et al., 2020). Those who completed the test session were allowed to participate in a lottery of two premium accounts, consisting of 6 months of free use on Hippocampus.

Altogether 185 students completed the test session to the end. However, as is common on MOOC platforms, the test takers were highly varying in terms of how much time they had been spent studying at Hippocampus. For leveling out those who only was visiting the platform from those that actually used the platform for studying, we followed the threshold criteria used in Fellman et al. (2020). First, we excluded participants that had been active less than 10 times during the first 100 days since registering themselves on the system (i.e., only one login session), resulting in the exclusion of 80 students. For the remaining participants (*N* = 105), we split the data into two groups with respect to retrieval practice activity as follows: those students that had completed ≥ 50 quizzes formed a group coined as *high retrieval practice group* (high-RP) whereas those that had completed <50 quizzes formed a separate group coined as *low retrieval practice group* (low-RP)^2^.

Together, these criteria resulted in a total sample size of 105 participants, with 56 of the participants belonging to the high-RP group and 49 of the participants to the low-RP group. As such, the participation rate was very low, considering that as many as 15,000 students are registered users. The mean age of the participants included in the present study was 30.29 years (*SD* = 7.06) out of which 49.52% were females. An independent samples *t*-test verified that the groups did not differ significantly in terms of age [*t*(104) = 0.682, *p* = 0.50], and there were no statistically significant differences between the two groups with respect to gender (χ2 = 9.153, *df* = 2.380, *p* = 0.67), and education (χ2 = 2.429, *df* = 4, *p* = 0.66). See also Table 1 that summarizes the demographical data of the participants.

**TABLE 1:** Background characteristics of the study sample.

	High-RP	Low-RP
Sample size (*n*)	56	49
Gender (F/M)	27/29	25/24
Age (M, SD)	29.80 (6.48)	30.80 (7.06)
Education	Basic vocational 12.5%	Basic vocational 8.16%
	Bachelor’s degree 25.0%	Bachelor’s degree 28.6%
	Master’s degree 55.4%	Master’s degree 59.2%
	Doctoral degree 3.6%	Doctoral degree 4.1%
	Other 3.6%	Other 0.0%

*High-RP = High retrieval practice group; Low-RP = Low retrieval practice group.*

### Target Outcomes of Retrieval Practice Activities

As previously mentioned, participants were prompted with optional quizzes following each study session at Hippocampus. These quizzes could be either in multiple-choice format or open-ended format. In the multiple choice quizzes, the participants were asked about specific information concerning the learning section followed by four alternatives out of which one was correct. Correctly recalled quiz responses were logged as ‘*True’* whereas incorrectly recalled quiz responses were logged as ‘*False.’* In the self-assessed quiz format, participants were prompted with a quiz in a similar fashion as in the multiple-choice quizzes. However, instead of being prompted with four alternatives, they were now asked to respond to the quiz in a written format by typing down their response in an empty box. Following the response, the system showed the correct answer. Thus, the scorings of the responses were self-corrected, meaning that the participants were to tick either on a red box with a text stating *Read more* (corresponding to an incorrectly recalled quiz and marked as *False* in the log file) or a green box with a text stating *I knew this* (corresponding to a correctly recalled quiz and marked as *True* in the log file).

We extracted three outcome variables from the Hippocampus platform that captured different aspects of retrieval practice activities: (1) *Number of taken quizzes per study session* (Quizzes per session), (2) *accuracy in taken quizzes* (Quiz performance), and (3) *processing speed in quizzes* (Quiz processing speed). Quizzes per session were calculated by averaging the number of taken quizzes (including both multiple-choice and self-assessed items) across all login sessions (formula: *Quizzes per session = total number of quizzes/total number of login sessions*). Quiz performance encompassed only the multiple-choice items (the self-assessed items were excluded as students could self-correct the responses *a posteriori*) and was calculated as a proportion score of correct responses (formula: Quiz performance = *number of correctly recalled quizzes/total number of completed quizzes*). Quiz processing speed comprised of the average time spent on a given quiz (formula: *Quiz processing speed = total quiz time/number of completed quizzes*).

#### Predictors of Individual Differences in Cognition and Personality

##### Raven’s advanced progressive matrices (RAPM)

For capturing cognitive ability, the participants were measured with Raven’s Advanced Progressive Matrices (RAPM) (Raven et al., 1991). In this task, 24 items were presented in ascending order (i.e., item difficulty increased progressively), each of which consisted of a 3 × 3 matrix of geometric patterns with the bottom-right area missing a pattern. The participants were asked to complete the pattern by picking one option among eight alternatives. The participants had 20 min to complete the task. As the dependent variable, we used the total number of correctly recalled items (score range 0–24), with higher scores indicating better reasoning ability. Internal consistency was good for RAPM in the present study (Cronbach’s α = 0.83).

##### Short grit scale-s (GRIT-S)

A Swedish version of the short version of GRIT (GRIT-S; Bertilsson et al., 2017) was used in the present study. GRIT-S includes eight items. Four of the items reflect participants’ ability to maintain interest (e.g., “I often set a goal but later choose to pursue a different one”) whereas the four other items capture participants’ ability to maintain effort (e.g., “I have achieved a goal that took years of work”). Each item is rated on a 5-point Likert-like scale (1 = strongly disagree 3 = neutral, and 5 = strongly agree). The scores from each individual item were averaged together and served as our dependent variable, with higher scores indicating more GRIT-S. Cronbach’s α for GRIT-S in the present study was 0.76, indicating acceptable internal consistency.

##### Need for cognition (NFC)

Need for cognition (NFC) was measured with the Mental Effort Tolerance Questionnaire (METQ; Stenlund and Jonsson, 2017), which is a Swedish adaptation of the original Need for Cognition Scale (Cacioppo and Petty, 1982). The NFC questionnaire encompasses 30 items, each of which is rated on a five-point Likert scale (1 = strongly disagree; 3 = neutral; 5 = strongly agree), yielding a possible score range from between 30 and 150. Twelve of the items represent positive attitudes toward engaging and enjoying thinking, whereas the remaining items indicate negative attitudes. Thus, the items capturing negative attitudes were reversed before calculating our dependent variable (i.e., the sum score of after all items were summed together), with higher scores indicating more NFC. Internal consistency was acceptable for NFC in the present study (Cronbach’s α = 0.75).

## Results

### Between-Group Analyses

First, we examined whether the low-RP individuals (*n* = 49) differed from the high-RP individuals (*n* = 56) with respect to our three predictors. We employed logistic regression analyses where the group served as the dependent variable and the predictor of interest as the independent variable. Moreover, number of login sessions served as the covariate in the models to control for activity effects (i.e., it is likely that those having more login sessions also have a higher probability of belonging to the high RP group). The results showed that, after controlling for number of study sessions (*p* < 0.001), RAPM had a statistically significant effect on group (β = 0.67, *p* = 0.011). Specifically, one unit increase in RAPM increased the odds ratio for being a high-RP individual with 1.18 (95% CI: 1.04–1.35). The personality predictors GRIT-S (β = −0.48, *p* = 0.592), and METQ (β = −0.11, *p* = 0.636) did not significantly predict group affiliation after controlling for number of login sessions.

### Within-Group Analyses of the High-RP Group

We further investigated how the three retrieval practice activities (i.e., quizzes per session, quiz performance, quiz processing speed) in the high RP group. Of note, we decided not to include the low-RP group in the within-group analyses, as the distribution in the three dependent variables of retrieval practice activities were highly non-normal. Specifically, most participants in the low-RP group had taken ≤ 1 quizzes, yielding unreliable results in the two other retrieval practice outcomes quiz performance (e.g., an individual with 1/1 correct quizzes obtains 100% accuracy) and quiz processing speed (e.g., quiz response time is calculated based on only one or a few items) as well.

We employed multiple regression analyses for investigating the relationship between the predictors and the retrieval practice variables. Specifically, this yielded three different models where a given retrieval practice variable was regressed on all predictors. Prior to analyses, the three retrieval practice measures were screened for multivariate outliers using the Mahalanobis distance value χ^2^ table (*p* < 0.001; Tabachnick and Fidell, 2007). We also screened each predictor variable (i.e., NFC, GRIT-S, RAPM) and dependent variable (i.e., retrieval practice activities) for univariate outliers (scores on any online activity feature that deviated more than 3.5 *SD* from the z-standardized group mean were defined as univariate outliers). All identified outliers from the aforementioned screening analyses were imputed using multivariate imputations by chained equations (MICE) (van Buuren and Groothuis-Oudshoorn, 2011). Following data cleaning, the assumptions for multiple regression (multicollinearity, homoscedasticity, multivariate normality, lack of outliers in standardized residuals) were met in all three models. Table 2 depicts descriptive statistics for the extracted retrieval practice activity variables and the three predictors, whereas zero-order correlations between the predictors and the retrieval practice variables can be found in Table 3. With respect to correlational relationships, we observed a statistically significant association between quizzes per session and quiz processing speed (*r* = −0.337, *p* = 0.012), between quiz performance and RAPM (*r* = 0.512, *p* < 0.001), between quiz processing speed and RAPM (*r* = 0.356, *p* = 0.007), and between RAPM and GRIT-S (*r* = −0.265, *p* = 0.048).

**TABLE 2:** Descriptive statistics for the extracted retrieval practice activity variables and the predictors.

Variable	M	*SD*	Skew	Kurtosis
Number of quizzes per session	27.13	16.79	1.12	0.54
Quiz performance	0.76^a^	0.11	–0.57	–0.1
Quiz processing speed	0.74^b^	0.24	0.34	–0.28
RAPM	18.79	4.33	–1.52	2.66
GRIT-S	3.30	0.62	–0.12	0.08
NFC	114.71	10.13	–0.38	–0.63

*^*a*^Proportion of correctly recalled quizzes; ^*b*^Depicted in minutes, *N* = 56.*

**TABLE 3:** Intercorrelations between the retrieval practice variables and the predictors.

Variable	1	2	3	4	5
					
1. Quizzes per session	−				
2. Quiz performance	0.16	−			
3. Quiz processing speed	−0.34*	–0.25	−		
4. RAPM	0.10	0.36**	−0.51**	−	
5. GRIT-S	0.14	0.06	–0.06	−0.27*	–
6. NFC	0.02	0.22	–0.17	0.21	0.10

*indicates *p* < 0.05, ** indicates *p* < 0.01.

#### Quizzes per Study Session

The regression model with quizzes per session as the dependent variable, and RAPM, GRIT-S and NFC as predictors was statistically non-significant [*F*(4, 52) = 1.472, *p* = 0.536, *R*^2^_Adjusted_ = −0.015]. A closer examination of the coefficients (see Table 4) showed that none of the predictors were significantly related to quizzes per session (all *p*-values ≥ 0.198).

**TABLE 4:** Regression coefficients with quizzes per study session as the outcome variable.

	*B*	*SE B*	β	*t*-value	Sig.
RAPM	0.593	0.565	0.153	1.05	0.298
GRIT-S	5.067	3.89	0.186	1.303	0.198
NFC	–0.051	0.234	–0.031	–0.218	0.828
*R*^2^_Adjusted_			–0.015		

*RAPM = Raven’s Advanced Progressive Matrices, GRIT-S = Short Grit Scale-S, NFC = Need for Cognition.*

#### Quiz Performance

When quiz performance served as the dependent variable, the predictors together explained 11.9% of the variance and the regression equation was statistically significant [*F*(4, 52) = 3.478, *p* = 0.022]. A closer inspection of the coefficients (see Table 5) showed that RAPM was significantly related to quiz performance (β = 0.368, *p* = 0.009) such that those with better reasoning performance having higher quiz performance scores. Neither GRIT-S nor NFC were significantly related to quiz performance (*p’*s ≥ 0.29).

**TABLE 5:** Regression coefficients with quiz performance as the outcome variable.

	*B*	*SE B*	β	*t*-value	Sig.
RAPM	0.009	0.003	0.368	2.711	0.009
GRIT-S	0.026	0.024	0.143	1.07	0.290
NFC	0.001	0.001	0.126	0.961	0.341
*R*^2^_Adjusted_			0.119*		

*RAPM = Raven’s Advanced Progressive Matrices, GRIT-S = Short Grit Scale-S, NFC = Need for Cognition.*

#### Quiz Processing Speed

In the regression model with quiz processing speed as the dependent variable, the results showed a statistically significant regression equation [*F*(4, 52) = 7.494, *p* < 0.001]. Together, the three predictors explained 26.2% of the variance in quiz processing speed. As depicted in Table 6, RAPM significantly predicted quiz processing speed (β = −0.559, *p* < 0.001), with those performing better in the reasoning task had faster quiz processing speed. Neither GRIT-S nor NFC were significantly related to quiz processing speed.

**TABLE 6:** Regression coefficients with quiz processing speed as the outcome variable.

	*B*	*SE B*	β	*t*-value	Sig.
RAPM	–0.032	0.007	-0.559	–4.506	<0.001
GRIT-S	–0.08	0.048	-0.202	–1.653	0.104
NFC	–0.001	0.003	-0.027	–0.22	0.827
*R*^2^_Adjusted_			0.262***		

*RAPM = Raven’s Advanced Progressive Matrices, GRIT-S = Short Grit Scale-S, NFC = Need for Cognition.*

### Follow-Up Analysis: Moderation Analyses

For examining whether the personality measures GRIT-S and NFC moderated the relationship between RAPM and retrieval practice activities, we followed up the previous analyses with moderation analyses. GRIT-S and NFC, which were fed into separate models with RAPM in these analyses, were transformed into binary variables using median splits prior to model computation (i.e., those with scores above median were defined as high GRIT-S/high NFC, whereas those having scores below the median were defined as low GRIT-S/low NFC). As we were interested in examining whether GRIT-S and NFC moderated the relationship between RAPM and each of the three retrieval practice activity variables, altogether six separate models were computed (for more information, see Supplementary Material). The results of the moderation analyses are summarised in Supplementary Material (Appendix A, Table A1), showing that neither personality variable moderated the relationship between RAPM and retrieval practice activities.

## Discussion

Retrieval practice is a well-established evidence-based study technique shown to have facilitating effects on long-term memory retention of information (Roediger and Karpicke, 2006; Butler, 2010; Weinstein et al., 2010; Agarwal et al., 2012), which have led several MOOC administrators to implement features tapping on retrieval practice on their platform. However, optionally based quizzes on MOOCs tend to be highly unutilized, and it is scarcely unknown which individuals, and in what way retrieval practice on in e-learning is used. This study set out to test how individual differences in cognition and personality is associated with retrieval practice activities on a MOOC platform targeted for university students. As a first step, we employed logistic regression analyses to examine whether low retrieval practice individuals (low-RP) differed from high retrieval practice individuals (high-RP) with respect to our three predictors tapping on reasoning (RAPM), and two personality measures capturing students ability to maintain interest over time (GRIT-S), and the tendency to engage in and enjoy thinking (NFC). As a second step, we conducted multiple regression analyses within the high-RP group where three relevant target outcomes of retrieval practice activities (number of taken quizzes per session, accuracy in quizzes, quiz processing speed) were regressed on our three predictors.

### Cognitive Ability and MOOC Retrieval Practice Activities

The results from the between-group analyses showed that fluid reasoning was a significant predictor for what group a given student belonged to after controlling for user activity. More specifically, a one point increase in the reasoning task increased the odds ratio of being a high-RP individual with 1.18. This finding aligns well with previous MOOC evidence, showing that cognitively high performing individuals typically tend to use optionally based quizzes more extensively than low-performing individuals on e-learning platforms (Fellman et al., 2020). Also in experimental settings, high-performing individuals typically use efficient study techniques (Barnett and Seefeldt, 1989), and strategies (Bailey et al., 2009) to a greater extent as compared with cognitively low-performing individuals.

The results from the within-group analyses showed that reasoning had a weak impact on the number of quizzes students took per reading session. Hence, this result is in discrepancy to the ones obtained from the between-group analysis. However, there might be other extraneous factors which potentially masks the true relationship between reasoning and quiz volumes in the latter analysis. First, our sample size was small in the within-group analysis, which increases the risk of making type II errors. Ideally, an inclusion of the low-RP group would both have increased the statistical power of the within-group analysis, and mimicked the between-group analysis to a greater extent, but as the participants in the low-RP group had barely engaged in retrieval practice activities on the MOOC platform, it prohibited us to include them in the analysis. Second, quizzing typically remains highly unutilized on MOOCs when the items remain only optionally available (Corral et al., 2020). Thus, it is also possible that quiz volume effects are difficult to observe when students merely use retrieval practice on MOOCs even if they have the possibility to do so.

**FIGURE 1:**
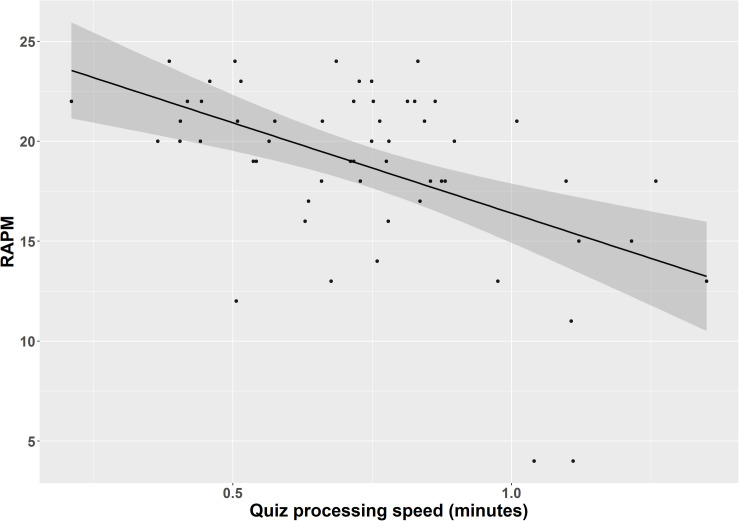
Regression plot with average processing speed (depicted in minutes) in quizzes on the x-axis and sum scores in the Raven’s Advanced Progressive Matrices (RAPM) on the y-axis. Shaded regions represent 95 % confidence intervals on the slope.

As regards quiz performance, the results from the within-group analysis showed that those with better reasoning abilities had quiz higher accuracy scores on the MOOC. This result aligns well with a body of experimental evidence, showing that high performing individuals typically have better recall performance in retrieval practice items (Tse and Pu, 2012; Agarwal et al., 2017; Minear et al., 2018). Moreover, those performing better in the reasoning task processed the quizzes on the MOOC platform more rapidly as compared to those with lower reasoning scores (see Figure 1). This result is supported with factor-analytical evidence, showing that cognitive abilities and processing speed are correlated, yet separable constructs (Conway et al., 2002; Martínez and Colom, 2009). Thus, the relationship observed here does not deviate from findings typically obtained in laboratory settings.

### Personality and MOOC Retrieval Practice Activities

Regarding the relationship between retrieval practice activities and our personality measures were generally weak. More specifically, between-group analyses showed that both NFC and GRIT-S did not significantly predict to which group individuals belonged to. Accordingly, the within-group analyses revealed that the personality measures were not significantly related to neither quizzes taken per session, quiz performance, nor quiz processing speed. Our follow-up analyses also showed that our personality measures had no moderating effects on the relationship between RAPM and retrieval practice activity, which further underscores their insignificant influence on how individuals engage in retrieval practice activities online. To our knowledge, it is rather unstudied how personality characteristics relate to quiz performance in retrieval practice items on MOOCs. The only comparable data stems from studies in experimental settings (Bertilsson et al., 2017; Stenlund et al., 2017), indicating retrieval practice accuracies are weakly related to individual differences in personality. Thus, although the relationship between personality and retrieval practice activities was weak in the present study, it both supports and extends existing research by showing that personality measures are weakly related to retrieval practice activities on e-learning platforms as well.

### Limitations

There are several limitations that should be regarded as shortcomings in the present study, and that could be addressed in a better way in future studies. First, the present study encompassed a relatively homogenous sample, mainly consisting of medical students, with the majority of them probably belonging to the most gifted individuals in the normal population. Thus, the generalization of the findings of the present study to other online groups should be interpreted with caution. Second, and partly related to the issue above, 185 participants out of a total of 15,000 students at Hippocampus took part in the study, with 105 participants included in the analyses. This is clearly a shortcoming, and which further underscores a tentative generalization of our results. Third, the study exhibited low statistical power, and thus the lack of effects, especially in the within-group analyses, can be questioned with respect to potential type II errors (Faber and Fonseca, 2014). The inclusion of the low-RP group would indeed have increased the sample size, but as mentioned earlier, their low engagement in retrieval practice activities on the MOOC platform prohibited us to include them in the analysis. Fourth, due to the lack of reliability and validity values of the retrieval practice activity variables that we extracted in this study, one can question what these outcome variables in fact capture. Although we cannot be entirely sure that each of them is tapping on relevant retrieval practice activities, we can, however, be confident that they at least measure different aspects of activity due to their relatively low intercorrelations with each other. Fifth, a shortcoming that pertains to all MOOCs is the lack of experimental control. The participants exhibited high independence when using the online platform, having the possibility to jump back and forth from a course to another, and complete courses and quizzes at their own pace. Future studies could assess the same research question as we did in the present study, yet with a more controlled user navigation and where participants receive identical stimuli during course completion.

### Conclusion and Future Directions

This study examined whether interindividual differences in cognitive ability, and personality characteristics were related to retrieval practice activities on a MOOC platform where students have the optional possibility to quiz themselves following each study session. Between-group analyses revealed that cognitively high performing individuals were more likely to use the optional quizzes on the platform. Moreover, within-group analyses including those students using the optional quizzes on the platform, showed that reasoning significantly predicted quiz performance, and quiz processing speed, but not number of quizzes. However, NFC and GRIT-S were unrelated to each of the aforementioned retrieval practice activities. From a more broad perspective, it appears that reasoning is a stronger predictor for retrieval practice usage on MOOCs as compared to self-assessed personality measures. Moreover, our results contribute to the research within teaching and learning across different environments in the digital age, by implicating that retrieval practice tend to be more used by cognitively high-performing individuals, bearing importance for MOOC administrators, especially from a personalization perspective (i.e., tailor-made learning in relation to students’ personal profiles).

Furthermore, we hope that our obtained results could serve as a framework for forthcoming studies that examines individual differences in cognition, personality together with other potentially relevant background factors, and how these relates to retrieval practice activities on MOOCs. One interesting topic for further studies could be to specifically elucidate how other personality measures, such as Openness and Conscientiousness from the “Big five” personality inventory (Goldberg et al., 2006), are related to retrieval practice activities on MOOCs.

## Data Availability Statement

The datasets and scripts for feature extraction and analyses will be made available by the authors upon request.

## Ethics Statement

This study involving human participants was reviewed and approved by the Regional Vetting Committee (2017/517-31), Sweden. The participants provided their written informed consent to participate in this study.

## Author Contributions

DF and BJ developed the study concept, conducted the feature extraction- and data preprocessing, performed the data analysis and interpretation, and drafted the manuscript. All coauthors provided critical revisions and approved the final version of the manuscript for submission and contributed to the study design.

## Conflict of Interest

The authors declare that the research was conducted in the absence of any commercial or financial relationships that could be construed as a potential conflict of interest.

## Footnotes

1 Besides the reasoning task and the questionnaires, participants also completed several other tasks tapping on working memory and episodic memory. However, results attributed to these tasks will be reported elsewhere.

2 After a careful exploratory data analysis, this threshold proved to be optimal based on two important criteria: (1) the median value of this variable was 58, thus very close to 50 completed quizzes used as cut-offs, and (2) this threshold proved to spread the participants to the respective groups fairly evenly.
