# Supplementary material for: Do Individual Differences in Cognition and Personality Predict Retrieval Practice Activities on MOOCs?
Source: Front Psychol. 2020 Aug 18;11:2076. doi: 10.3389/fpsyg.2020.02076 (PMC7461909; doi:10.3389/fpsyg.2020.02076)
Supplement: Supplementary file 1 [file Table_1.DOCX]

**APPENDIX A**

A total of six moderator analyses were computed. These models, differing with respect to the dependent variable and moderator of interest, were as follows:

- **Model 1**: *quizzes per session* served as the dependent variable, whereas main effects of RAPM and GRIT-S, together with their interaction term served as the predictors.
- **Model 2**: *quizzes per session* served as the dependent variable, whereas main effects of RAPM and NFC together with their interaction term served as the predictors.
- **Model 3**: *quiz performance* served as the dependent variable, whereas main effects of RAPM and GRIT-S, together with their interaction term served as the predictors.
- **Model 4**: *quiz performance* served as the dependent variable, whereas main effects of RAPM and NFC, together with their interaction term served as the predictors.
- **Model 5**: *quiz processing speed* served as the dependent variable, whereas main effects of RAPM and GRIT-S together with their interaction term served as the predictors.
- **Model 6**: *quiz processing speed* served as the dependent variable, whereas main effects of RAPM and NFC together with their interaction term served as the predictors.

Note that in each model, the interaction between RAPM and the personality measures is of most interest, as it reveals whether the relationship between RAPM and retrieval practice activity is moderated by the personality measure. The results from the six models are presented in Table A1.

| **Table A1.** Moderation analyses | | | | | |
| --- | --- | --- | --- | --- | --- |
|  | *B* | *SE B* | β | t-value | Sig. |
|  | **Model 1 (Quizzes per session)** | | | | |
| RAPM | 1.029 | 1.096 | 0.265 | 0.939 | 0.352 |
| GRIT-S | 8.449 | 4.558 | 0.253 | 1.853 | 0.07 |
| RAPM × GRIT-S | -2.753 | 5.405 | -0.142 | -0.509 | 0.613 |
|  | **Model 2 (Quizzes per session)** | | | | |
| RAPM | -0.15 | 0.77 | -0.039 | -0.195 | 0.846 |
| NFC | 0.46 | 4.707 | 0.014 | 0.098 | 0.923 |
| RAPM × NFC | 4.518 | 4.748 | 0.186 | 0.952 | 0.346 |
|  | **Model 3 (Quiz performance)** | | | | |
| RAPM | 0.016 | 0.007 | 0.608 | 2.253 | 0.029 |
| GRIT-S | 0.024 | 0.029 | 0.111 | 0.853 | 0.398 |
| RAPM × GRIT-S | -0.034 | 0.034 | -0.265 | -0.992 | 0.326 |
|  | **Model 4 (Quiz performance)** | | | | |
| RAPM | 0.011 | 0.005 | 0.426 | 2.277 | 0.027 |
| NFC | -0.008 | 0.029 | -0.037 | -0.276 | 0.784 |
| RAPM × NFC | -0.014 | 0.03 | -0.085 | -0.461 | 0.647 |
|  | **Model 5 (Quiz processing speed)** | | | | |
| RAPM | -0.047 | 0.014 | -0.824 | -3.372 | 0.001 |
| GRIT-S | -0.077 | 0.057 | -0.158 | -1.338 | 0.187 |
| RAPM × GRIT-S | 0.092 | 0.068 | 0.326 | 1.348 | 0.184 |
|  | **Model 6 (Quiz processing speed)** | | | | |
| RAPM | -0.031 | 0.009 | -0.545 | -3.295 | 0.002 |
| NFC | -0.115 | 0.057 | -0.237 | -2.005 | 0.050 |
| RAPM × NFC | 0.046 | 0.058 | 0.131 | 0.803 | 0.426 |
| Note. RAPM = Raven’s Advanced Progressive Matrices, GRIT-S = Short Grit Scale-S, NFC = Need for Cognition. | | | | | |
